# Draft Genome Sequence of Purpureocillium takamizusanense, a Potential Bioinsecticide

**DOI:** 10.1128/mra.00268-22

**Published:** 2022-06-28

**Authors:** Ngoc-Hung Nguyen, Tomokazu Tamura, Kiminori Shimizu

**Affiliations:** a Department of Biological Science and Technology, Tokyo University of Science, Katsushika, Tokyo, Japan; b Medical Mycology Research Center, Chiba University, Chuo-ku, Chiba, Japan; Vanderbilt University

## Abstract

To investigate the biocontrol capability of the entomopathogenic fungus Purpureocillium takamizusanense, the genome of the wild-type strain isolated from synnemata on Meimuna opalifera, was sequenced using a combination of HiSeq and Nanopore technologies, and annotated using evidence from RNA sequences and protein sequences from its sister species Purpureocillium lilacinum.

## ANNOUNCEMENT

Purpureocillium takamizusanense (phylum Ascomycota, class Sordariomycetes, order Hypocreales, family Ophiocordycipitaceae) is a species related to the better-known biocontrol agent Purpureocillium lilacinum (1). To investigate the capability of *P. takamizusanense* as a bioinsecticide, the complete genome of this species was sequenced, assembled, and annotated.

The wild-type strain (PT3) of *P. takamizusanense* was isolated from synnemata on Meimuna opalifera collected in Chiba, Chiba Prefecture, Japan (35°34′47.9″N, 140°13′39.0″E), on 7 August 2016. The isolated strain was grown on potato dextrose broth (BD Difco) at 25°C with shaking at 120 rpm for 5 days (for DNA extraction) and 7 days (for RNA extraction). Genomic DNA was extracted using the DNAeasy plant minikit (Qiagen) following the fungal DNA isolation protocol. For Illumina sequences, a library was constructed using the Nextera DNA library preparation kit and sequenced on a HiSeq X instrument using the paired-end 150-bp protocol. For the Nanopore platform, DNA was prepared using the ligation sequencing kit (SQK-LSK109) and sequenced on the PromethION platform using FLO-PRO002 flow cells. RNA was extracted using the RNA premium kit (FastGene), a sequencing library prepared using the TruSeq stranded mRNA kit, and sequenced using the Illumina NovaSeq instrument. Finally, we obtained 44,316,524 HiSeq reads (~3.51 Gb), 407,870 long reads (~4.09 Gb; mean, 10.92 kb; read *N*_50_, 17,299 kb; and longest read, 55,422 kb), and 98,646,618 RNA reads (~14.896 Gb). The Nanopore reads were adapter trimmed using Porechop v0.2.4 (https://github.com/rrwick/Porechop), contamination filtered using NanoLyse v1.1.0 (2), and size filtered (>1,000 bp) using SeqKit v2.2.0 (3).

The draft genome was first assembled using the MaSuRCA pipeline (4) with both the HiSeq and Nanopore sequences. The assembly was polished by mapping the HiSeq sequences to the assembly using BWA-MEM v0.7.17 (5) and then implementing four rounds of polishing using Pilon v1.24 (6). A mitochondrial contig was identified by mapping the polished assembly against the mitochondrial genome of *P. lilacinum* (7) (GenBank accession no. MN635609) using Minimap2 v2.22 (8); it was extracted, and the overlapping ends of the circular sequence were trimmed using seq-circ-trim (9). The gene annotation was conducted using MFannot (10) and the MITOS (11) Web server (based on Genetic Code 4) and checked visually and combined manually using the Integrative Genomics Viewer (IGV) (12). Repeated sequences of the assembled genome were predicted using RepeatModeler v2.0.1 (13) and marked using RepeatMasker v4.1.1 (14). The RNA sequences were first adapter cut using Cutadapt v1.15 (15) and then mapped and indexed to repeat the masked assembly using HISAT2 (16) and SAMtools v1.15 (17). Gene prediction and annotation were performed using a protein homology search with the most closely related species, *P. lilacinum* (GCA_001653265), and RNA-seq using the Funannotate v1.8.1 pipeline (18), respectively. The completeness of the annotation was accessed using BUSCO v4.1.3 with 3,817 ortholog genes from the class Sordariomycetes (19). For the chromosome scaffolds, a total of 11,855 protein-coding genes were predicted (18) (Table 1). The complete mitochondrial genome of *P. takamizusanense* is a closed circular molecule of 33,113 bp thats contains 61 genes (Table 1). For the analyses in this study, default parameters were used except where otherwise noted.

**TABLE 1:** Genome features of the *Purpureocillium takamizusanense* assembly and annotation

Characteristic	Value
Chromosome scaffolds	
Total assembly length (Mb)	35.6
No. of scaffolds	14
*N*_50_ (Mb)	3.09
*L*_50_	4
Max length (Mb)	6.1
No. of complete BUSCOs^*a*^ (%)	3,791 (99.3)
No. of incomplete BUSCOs (%)	12 (0.3)
No. of missing BUSCOs (%)	14 (0.4)
% repeats	4.6
%GC	57.76
No. of protein-coding genes	11,855
No. of tRNAs	85
No. of Phobius secretome genes	1,089
No. of Phobius transmembrane proteins	2,680
No. of antiSMASH biosynthetic gene clusters	36
Mitochondrial genome	
Length (bp)	33,113
No. of protein-coding genes	24
No. of rRNAs	2
No. of tRNAs	35

a BUSCOs, benchmarking universal single-copy orthologs.

### Data availability.

The *Purpureocillium takamizusanense* genome project, including the raw Illumina, Nanopore, and RNA data, has been deposited at DDBJ/EMBL/GenBank under BioProject accession number PRJNA685267. The assembled nuclear genome and assembled mitochondrial sequences are available under GenBank accession numbers GCA_022605165.1 and OK505612, respectively. The raw HiSeq, Nanopore, and RNA sequencing reads have been deposited at the Sequence Read Archive under accession numbers SRR16282004, SRR16282003, and SRR16888993, respectively.
